# Influenza A matrix protein M1 induces lipid membrane deformation via protein multimerization

**DOI:** 10.1042/BSR20191024

**Published:** 2019-08-05

**Authors:** Ismail Dahmani, Kai Ludwig, Salvatore Chiantia

**Affiliations:** 1 University of Potsdam, Institute of Biochemistry and Biology, Karl-Liebknecht-Str. 24-25, Potsdam 14476, Germany; 2 Forschungszentrum für Elektronenmikroskopie und Gerätezentrum BioSupraMol, Institut für Chemie und Biochemie, Freie Universität Berlin, Fabeckstr. 36a, Berlin 14195, Germany

**Keywords:** confocal microscopy, influenza, lipid membranes, membranes, protein-protein interactions, viral matrix proteins

## Abstract

The matrix protein M1 of the Influenza A virus (IAV) is supposed to mediate viral assembly and budding at the plasma membrane (PM) of infected cells. In order for a new viral particle to form, the PM lipid bilayer has to bend into a vesicle toward the extracellular side. Studies in cellular models have proposed that different viral proteins might be responsible for inducing membrane curvature in this context (including M1), but a clear consensus has not been reached. In the present study, we use a combination of fluorescence microscopy, cryogenic transmission electron microscopy (cryo-TEM), cryo-electron tomography (cryo-ET) and scanning fluorescence correlation spectroscopy (sFCS) to investigate M1-induced membrane deformation in biophysical models of the PM. Our results indicate that M1 is indeed able to cause membrane curvature in lipid bilayers containing negatively charged lipids, in the absence of other viral components. Furthermore, we prove that protein binding is not sufficient to induce membrane restructuring. Rather, it appears that stable M1–M1 interactions and multimer formation are required in order to alter the bilayer three-dimensional structure, through the formation of a protein scaffold. Finally, our results suggest that, in a physiological context, M1-induced membrane deformation might be modulated by the initial bilayer curvature and the lateral organization of membrane components (i.e. the presence of lipid domains).

## Introduction

The Influenza A virus (IAV) buds from the plasma membrane (PM) of infected cells and, while doing so, acquires a lipid envelope from its host [1–4]. During this step, the PM lipid bilayer is initially bent into a vesicle, toward the extracellular side [5]. Assembly of viral components, bending of the lipid bilayer and the resulting budding of virions are essential parts of the IAV replication cycle and, therefore, their regulation could be a potential target for antiviral therapies.

IAV assembly is thought to be orchestrated by its matrix protein M1, which mediates the interactions among other viral components and the lipids in the PM [6]. In spite of its fundamental importance, this process is still not fully understood. We and others have investigated M1–M1 and M1–lipid interactions, both in model membranes and cellular systems [7–9]. It was shown that M1 interacts electrostatically via its N-terminal domain with acidic lipids and this interaction modulates protein multimerization [7,9,10]. On the other hand, the process by which M1 or other viral proteins can induce membrane curvature during IAV budding is less clear. This issue has been investigated *in cellulo* without reaching a clear consensus [11]. In some cases, it was demonstrated that M1 alone is sufficient to induce budding at the PM, without the need for other viral components [12,13]. Other studies have shown instead that IAV hemagglutinin and neuraminidase are needed to produce viral particles, while M1 might not play a significant role [14–16]. Another group suggested that, in the presence of significant M1–PM binding, the protein might be capable of inducing budding [17]. In light of these observations, it would be therefore very interesting to investigate M1–lipid interactions in a controlled environment and clarify whether the protein has indeed the ability to induce curvature in lipid bilayers on its own. In this context, giant unilamellar vesicles (GUVs) provide a simplified model for the PM, consisting of a non-supported lipid bilayer with well-defined composition and physical properties [18–21]. GUVs have been used in the past to investigate the interplay between viral proteins and lipid membranes and, specifically, protein-induced alterations in the three-dimensional structure of the bilayer [22–25]. In this work, we used a similar approach to investigate the interaction between IAV M1 and lipid membranes, using a combination of fluorescence microscopy imaging, scanning fluorescence correlation spectroscopy (sFCS) [26], cryogenic transmission electron microscopy (cryo-TEM), as well as cryo-electron tomography (cryo-ET). Our results indicate that M1 is capable of causing membrane deformation, also in the absence of other proteins. Furthermore, we quantitatively demonstrate that M1 multimerization, rather than just binding, is necessary for the induction of membrane curvature.

## Materials and methods

### Chemicals

All lipids (i.e. dioleoyl-sn-glycero-3-phosphocholine (DOPC), 2-distearoyl-sn-glycero-3-phosphocholine (DSPC), 1,2-dioleoyl-sn-glycero-3-phospho-l-serine (DOPS), phosphatidylinositol 4,5-bisphosphate (PIP2), 1,2-dipalmitoyl-sn-glycero-3-phospho-(1′-rac-glycerol) (DPPG), cholesterol and 1,2-dioleoyl-*sn*-glycero-3-phosphoethanolamine-*N*-Lissamine rhodamine B sulfonyl (Rhodamine*-*DOPE)) were obtained from Avanti Polar Lipids (Alabaster, Alabama, U.S.A.). Alexa Fluor 488 succinimidyl ester was obtained from Life Technologies (Darmstadt, Germany). 2-(4-(3-(4-acetyl-3-hydroxy-2-propylphenoxy) propoxy) phenoxy acetic acid (PHE) was purchased from Cayman Chemical (Ann Arbor, MI, U.S.A.). Ten-fold concentrated phosphate buffer (PBS, 100 mM phosphate buffer, 27 mM potassium chloride and 1.37 M sodium chloride at pH 7.4) was from Alfa Aesar (Haverhill, MA, U.S.A.). Glucose was from VWR Chemicals (Radnor, PE, U.S.A.). All other chemicals were purchased from ROTH (Karlsruhe, Germany), unless differently specified. Indium tin oxide (ITO)-coated coverslips, 20 × 20 mm, thickness #1, 8–12 Ohms resistivity, were purchased from SPI supplies (West Chester, PA, U.S.A.).

### Preparation of GUVs

GUVs were prepared using the electroformation method [27,28]. Different lipid compositions were used to prepare the vesicles, as specified in each case in the ‘Results’ section. Typically, DOPC and cholesterol were mixed with various amounts of negatively charged lipids (e.g. DOPS) at molar ratios between 10 and 50 mol%. Rhodamine-DOPE 0.05 or 0.1 mol% was added as a tracer to allow GUV membrane visualization by confocal microscopy. The GUV electroformation chamber consisted of two conductive ITO coverslips facing each other and separated by a 3-mm thick Teflon spacer. The total volume of the chamber was ∼300 μl. Thirty microliters of 3 mM lipid solution in chloroform and/or ethanol were spread on a preheated ITO coverslip, forming a thin lipid film. The solvent was evaporated using a nitrogen flow for 5 min at room temperature (note that solvent evaporation under vacuum for 1 h did not show a difference in vesicle behavior). After assembly, the chamber was filled with a sucrose solution in deionized water (e.g. 150 mM) and connected to a voltage generator (AC generator FG 250 D, H-Tronic, Hirschau, Germany). For experiments at low pH, the chamber was filled with 150 mM sucrose, 10 mM acetate buffer solution at pH 5. A sinusoidal electric field of 1.4 V at 10 Hz was applied for 1.5 h at room temperature. To facilitate the detachment of GUV from the slides, the voltage was decreased to 0.5 V for 30 min.

### Preparation of GUVs showing phase separation

Vesicles were prepared as described in the previous paragraph, using a lipid mixture consisting of 10 mol% cholesterol, 45 mol% DOPC, 15 mol% DPPG and 30 mol% DSPC. Additionally, 0.05 mol% Rhodamine*-*DOPE was added to visualize the different lipid domains. Lipids were dissolved in chloroform/methanol 9:1 v:v (5 mM, prepared freshly and kept under a nitrogen atmosphere). The obtained lipid film was subjected to the electroformation procedure (see previous paragraph) at 50°C for 1.5 h. The chamber was then cooled down slowly at room temperature before imaging.

### Expression and purification of recombinant matrix protein (M1) constructs

6xHis-tag M1 protein from Influenza A/FPV/Rostock/34 was expressed and purified, using a protocol adapted from Hilsch et al. [9]*.* Rosetta *Escherichia coli* (DE3) pLysS-competent cells were grown in 1 l medium (containing 0.2% glucose, 50 mg/ml chloramphenicol and 50 mg/ml ampicillin) until reaching OD_600_∼0.7 at 37°C. Then, protein expression was induced by addition of IPTG (0.4 mM) while shaking for 3 h at 37°C. Bacteria were harvested by centrifugation for 10 min at 4800 rpm (Thermo Lynx 4000 F12-6 rotor, Thermo Fisher Scientific, Waltham, MA, United States). The obtained pellets were stored at −80°C until needed. On a different day, pellets were resuspended in lysis buffer (16 mM Na_2_HPO_4_, 146 mM KH_2_PO_4_, 500 mM NaCl, 5.4 mM KCl, EDTA-free protease inhibitor cocktail, 1 mM PMSF, 200 μg/ml DNAse), quickly frozen (15 min at −80°C) and incubated on a rotation shaker at 4°C for 30–60 min. All the following steps were performed at 4°C. The bacteria were completely lysed using a French press (Glen Mills, NJ, U.S.A.) run at 1000 psi. Finally the obtained bacterial lysate was clarified by centrifugation at 21000 rpm for 30 min (Thermo Lynx 4000, A27*-*8 rotor, Thermo Fisher Scientific, Waltham, MA, United States). The supernatant was incubated with 2 ml TALON resin (Takara, Saint-Germain-en-Laye, France) in a tube using a rotation shaker for 30–60 min. The resin was then washed with an equilibration buffer (8 mM Na_2_HPO_4_, 1.5 mM KH_2_PO_4_, 500 mM NaCl, 2.7 mM KCl, pH 7.4) and incubated on shaker for 15 min*.* The resin was washed again two to three times with an intermediate washing buffer consisting of the equilibration buffer with additional 60 mM imidazole, pH 7.2. Finally, M1 was eluted with an elution buffer (two-fold concentrated PBS with additional 180 mM imidazole, pH 7.4). Typical (maximal) protein concentrations were ∼60 μM. SDS/PAGE was performed to verify protein purity. Protein concentration was determined using UV absorbance at 280 nm on an Agilent 8453 UV/Vis spectrophotometer (Agilent Technologies, Milford, MA, U.S.A.).

For the experiments performed at pH 5, the protein was purified directly at low pH. In particular, the intermediate washing buffer contained 20 mM imidazole (pH 6.5). The elution buffer contained 50 mM sodium acetate and 300 mM NaCl (pH 5).

M1-derived constructs (i.e. N-terminal domain 1–164 aa (NM1) and C-terminal domain 165–252 aa (CM1)) were purified at pH 7.4 as previously described [10]. The elution buffer used for NM1 was the same as the one used for M1. The elution buffer used for CM1 was PBS with additional 150 mM imidazole (pH 7.4).

### Protein labeling for fluorescence microscopy

The purified protein was conjugated, if needed, with the primary amine-reactive dye Alexa Fluor 488 succinimidyl ester. Freshly purified protein (in elution buffer) was incubated with the reactive dye (10:1 molar ratio) for 2 h at 4°C, at the desired pH (7.4 or 5).

For simple imaging experiments, the protein–dye mixture was directly used, without further separation, as described in the next paragraph. For quantitative experiments (e.g. sFCS experiments), the labeled protein (M1-Alexa488) was separated from the free dye using size-exclusion chromatography (PD-10, GE Healthcare, Munich, Germany). The labeled protein was eluted with diluted PBS (pH 7.4), matched to the desired final osmolarity, as described in the next paragraph. Protein concentration and labeling efficiency were determined by absorbance at 280 and 490 nm, respectively. The maximum protein concentrations were typically ∼40 μM, while labeling efficiencies ranged between 0.1 and 0.15 dye/protein.

### Protein–GUV samples and imaging

**Figure 1:**
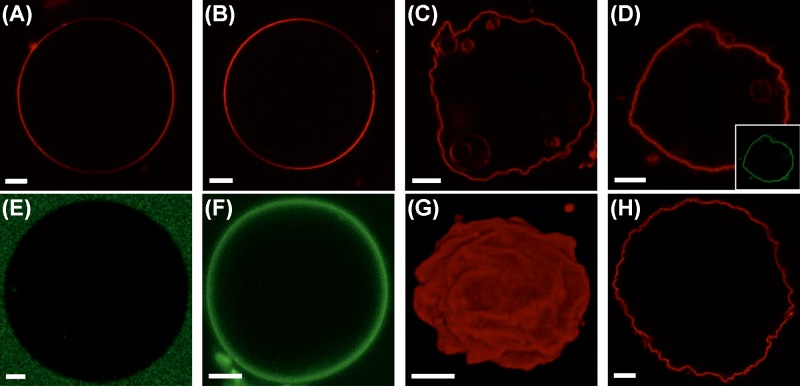
Shape alterations induced by M1 in DOPS-containing GUVs (**A**–**D**) Typical GUVs composed of 20 mol% Cholesterol, DOPC and increasing amounts of DOPS ((A) 0 mol%, (B) 10 mol%, (C) 30 mol%, (D) 50 mol%) observed via confocal LSM after 30-min incubation with 5 μM M1-Alexa488. Rhodamine-DOPE (0.01 mol%, red channel) was added to allow the visualization of the lipid bilayer via confocal LSM. **Inset in** (**D**–**F**): Fluorescence signal originating from M1-Alexa488 (green channel) for typical GUVs containing 0 mol% DOPS ((E) corresponding to the sample represented in (A)), 10 mol% DOPS ((F) corresponding to the sample represented in (B)) and 50 mol% DOPS (inset in (D)). (**G**) Three-dimensional reconstruction of a typical GUV containing 30 mol% DOPS in the presence of 5 μM M1-Alexa488 (corresponding to the sample represented in (C)). The fluorescence signal originates from Rhodamine-DOPE (0.01 mol%, red channel). (**H**) Confocal LSM image of a typical GUV composed of 30 mol% DOPC, 30 mol% DOPS and 40 mol% cholesterol, in the presence of 5 μM M1-Alexa488. The fluorescence signal originates from Rhodamine-DOPE (0.01 mol%, red channel). All GUVs contained 150 mM sucrose in their lumen and were suspended in a phosphate buffered protein solution (NaCl ∼25 mM, pH 7.4) with similar osmolarity (see ‘Materials and methods’ section). Scale bars are 5 μm. Images were acquired at 23°C. Abbreviation: LSM, laser scanning microscopy.

Before mixing with GUVs, the protein solution (i.e. M1 in elution buffer) was diluted with deionized water to reach the target approximate osmolarity. For example, for experiments in which GUVs were prepared in 150 mM sucrose (see experiments shown in Figures 1, 3 and 4), the purified M1 solution (with or without Alexa Fluor 488 succinimidyl ester) was diluted approximately five-fold. Typical protein concentrations at this step were ∼12 μM. The protein solution was then mixed with the GUV suspension with different volume ratios, in order to obtain the final desired protein concentration (usually between 5 and 10 μM) in a total of 300 μl volume, and transferred to 35- mm dishes (CellVis, Mountain View, CA) with optical glass bottoms, previously passivated with a 1% bovine serum albumin solution. This procedure ensured that sugar concentration and osmotic pressure across GUV membranes were always reproducible, at the expense of slight variations in buffer composition (e.g. NaCl between ~25 and ~70 mM, as indicated in each figure caption). It is worth mentioning that no significant alteration in M1–lipid interactions was reported in the presence of NaCl between 0 and 150 mM [7].

For the experiments carried in the presence of the M1-multimerization inhibitor PHE, M1 (∼60 μM) was incubated with PHE (100 μM, unless differently stated) for 30 min directly in the elution buffer. Subsequently, the mixture was incubated with 4 μM Alexa Fluor 488 succinimidyl ester, for 2 h at 4°C. Finally, the protein solution was diluted with deionized water to approximatively match the osmolarity of the vesicle suspension and mixed with the GUVs, so to obtain a final protein concentration of 10 μM (and ∼25 μM PHE).

Imaging experiments at pH 5 were carried out as follows: M1 was purified and labeled as described in ‘Expression and purification of recombinant matrix protein (M1) constructs’ and ‘Protein labeling for fluorescence microscopy’ sections directly at pH 5. The protein-dye solution in elution buffer (50 mM sodium acetate and 300 mM NaCl, pH 5) was subsequently diluted approximately three-fold using deionized water, to a final protein concentration of ∼13 μM (in ∼16 mM sodium acetate, ∼90 mM NaCl). This solution was finally mixed with the GUV suspension, so as to obtain a final protein concentration of 10 μM.

We have verified that the presence of imidazole does not affect membrane curvature for at least 2 h and that Alexa Fluor 488 succinimidyl ester does not significantly interact with GUVs (data not shown). Nevertheless, in the case of samples that required higher protein concentrations (i.e. >10 μM M1) or extensive removal of free dye, the elution buffer was exchanged after protein labeling, using a PD-10 column. For example, for the sFCS experiments described in ‘M1 exhibits reduced dynamics and high degree of multimerization in deformed vesicles’ section, the protein was eluted using two-fold diluted PBS (pH 7.4), to approximatiely match the osmolarity of GUV samples prepared in 150 mM sucrose.

Confocal fluorescence laser scanning microscopy (LSM) imaging was performed on a Zeiss LSM780 system (Carl Zeiss, Oberkochen, Germany) using a 40× 1.2 NA water-immersion objective. Samples were excited with a 488-nm argon laser (for the fluorophores Alexa Fluor 488 succinimidyl ester) or a 561-nm diode laser (for the fluorophore Rhodamine*-*DOPE). For measurements performed using 488  nm excitation, fluorescence was detected between 499 and 695 nm, after passing through a 488-nm dichroic mirror, using GaAsP detectors. For measurements performed with 561 nm excitation, fluorescence emission was separated using a 488/561 nm dichroic mirror and was detected between 570 and 695 nm.

ImageJ (NIH, Bethesda, MA, U.S.A.) was used to analyze microscopy images and determine the circularity of randomly selected GUVs. Vesicles with a circularity <0.85 were defined as deformed/non-spherical.

### Liposome preparation for cryo-TEM

Large unilamellar vesicles (LUVs) were prepared by extrusion. The desired lipids mixture (DOPC + 40 mol% DOPS) was dissolved in chloroform and then evaporated under nitrogen stream at room temperature to form a lipid film. Films were then rehydrated in PBS (pH 7.4). The obtained dispersion (2 mM total lipid concentration) was vigorously vortexed for 2–5 min. The diameter of the obtained multilamellar vesicles was reduced by serially extruding the suspension 11-times through a 100-nm pore diameter polycarbonate membrane (Whatman, Maidstone, U.K.) with a hand-held extruder (Avanti Polar Lipids, AL, U.S.A.). Liposome size (∼130 nm diameter in average) was measured via dynamics light scattering, using a Zetasizer HS 1000, (Malvern, Worcestershire, U.K.). The purified M1 (unlabeled) in elution buffer was diluted with deionized water to approximatively match the osmolarity of the liposome suspension (∼2.6-fold dilution). LUVs and protein solution were mixed in a total volume of 800 μl so to obtain a 10-μM final M1 concentration. Typical final lipid concentrations were ∼0.5 to 1 mM. Perforated carbon film-covered microscopical 200 mesh grids (R1/4 batch of Quantifoil, MicroTools GmbH, Jena, Germany) were cleaned with chloroform and hydrophilized by 60 s glow discharging at 8 W in a BAL-TEC MED 020 device (Leica Microsystems, Wetzlar, Germany), before 5 μl aliquots of the liposome/protein solution were applied to the grids. The samples were vitrified by automatic blotting and plunge freezing with a FEI Vitrobot Mark IV (Thermo Fisher Scientific, Waltham, U.S.A.) using liquid ethane as cryogen.

### Cryo-TEM

The vitrified specimens were transferred under liquid nitrogen into the autoloader of a FEI TALOS ARCTICA electron microscope (Thermo Fisher Scientific, Waltham, U.S.A.). This microscope is equipped with a high-brightness field-emission gun (XFEG) operated at an acceleration voltage of 200 kV. Micrographs were acquired on a FEI Falcon 3 4k × 4k direct electron detector (Thermo Fisher Scientific, Waltham, U.S.A.) using a 70-μm objective aperture at a primary magnification of 28 or 45 k, corresponding to a calibrated pixel size of 3.69 or 2.29 Å/pixel, respectively.

### Cryo-ET

Single-axis tilt series (±60° in 2° tilt angle increments) was recorded with the Falcon 3 direct electron detector at full resolution (28 K primary magnification) with a total dose lower than 70 e^−^/Å^2^. Tomogram reconstruction was performed using Thermo Fisher Inspect3D software. Amira, Version 6.0 (FEI, Oregon, U.S.A.) was used for visualization.

### sFCS

sFCS measurements were performed on a Zeiss LSM780 system using a 40× 1.2 NA water- immersion objective. M1-Alexa488 was excited with a 488-nm argon laser. Fluorescence was detected after passing through a 520/35 nm bandpass filter. Data acquisition and analysis were performed as described by Dunsing et al. [26,29]. Briefly, line scans of 128 × 1 pixels (pixel size 160 nm) were performed perpendicular to the GUV membrane with a 472.73 μs scan time. Typically, 600000 lines were acquired (total scan time ∼4 min) in photon counting mode. Low laser power was used (∼1 μW) to avoid photobleaching and fluorescence saturation effect. Data were exported as TIF files, and then imported and analyzed in Matlab (MathWorks, Natick, MA) using custom-written code [26,29]. The analysis thus obtained auto-correlation curves with a two-dimensional diffusion model resulted in the determination of the number of fluorophores in the confocal volume (N) and their apparent diffusion times. Furthermore, total fluorescence intensity at the GUV membrane and M1-Alexa488 brightness were determined. Protein brightness and fluorescence intensity were normalized to account for day-to-day variations in laser power, optics alignment and degree of protein labeling, as described in Hilsch et al. [9]. An approximate conversion from normalized brightness into the size of protein multimers is discussed in ‘M1 exhibits reduced dynamics and high degree of multimerization in deformed vesicles’ section, assuming for the sake of simplicity that the lowest measured average brightness value (∼0.025 a.u., sample ‘pH 5’) corresponds to M1 dimers [30] and using the formula described in Dunsing et al. [31], with pf = 0.1 and ε = 0.0227.

Statistical significance of differences among sFCS datasets were determined using a two- sided *t* test with distinct variances (ttest2 routine, Matlab).

## Results

### M1 induces membrane deformation in GUVs containing negatively charged lipids

In order to clarify whether M1 is sufficient to induce membrane deformation in protein-free lipid bilayers, we incubated GUVs of different compositions in the presence of 5 μM M1-Alexa448. It is worth noting that approximately one in ten proteins were labeled (see ‘Materials and Methods’ section). Freshly prepared lipid vesicles included increasing amounts of DOPS (from 0 to 50 mol%), as it was shown that this lipid promotes M1 binding via electrostatic interactions [7–10,32]. GUVs were stained with trace amounts of a fluorescent lipid (Rhodamine-DOPE) and observed via confocal LSM. According to Figure 1A–D,G, significant membrane deformation was observed for GUVs containing high amounts of DOPS (i.e. ≥30 mol%). GUVs containing low amounts of DOPS (e.g. 10 mol%, Figure 1B) or in control samples without protein (Supplementary Figure S1A) remained mostly spherical. We did not observe significant changes in the shape of deformed GUVs during the measurement time.

Although alterations in membrane shape could be obtained also by using non-fluorescent M1 (Supplementary Figure S1B), labeling of M1 was instrumental to directly visualize protein binding and organization. Figure 1E,F and the inset in panel (D) show the spatial distribution of M1-Alexa488 typically observed in the samples represented in panels (A,B,D), respectively. As expected, in the absence of DOPS, very little binding of M1 to the membrane was observed and most of the protein could be found in solution outside the GUVs (Figure 1E). In the case of GUVs containing 10 mol% DOPS, M1 bound homogeneously to the lipid membrane (Figure 1F). M1 binding appears therefore necessary but, in general, not sufficient to induce membrane deformation. Interestingly, we noticed that the protein bound homogeneously to non-spherical GUVs as well (e.g. Figure 1D and inset). Also, the protein fluorescence intensity inside and outside deformed GUVs was, in general, not distinguishable (see e.g. inset in Figure 1D). This indicates that M1 might have crossed the membrane into the lumen of the vesicles in many cases. Nevertheless, we observed that the shape of deformed GUVs was qualitatively reproducible also whenever M1 was more clearly excluded from the lumen of the vesicles (see Supplementary Figure S1B,C).

Additionally, an increase in membrane acyl-chain order (by increasing cholesterol concentration to 40 mol%, Figure 1H) did not appear to suppress membrane deformation. GUV shape alterations could also be observed if DOPS was substituted by other negatively charged lipids, e.g. PIP2 or PG (data not shown). Finally, we observed that the N-terminal domain of M1 (M1N aa. 1–164) is sufficient to induce membrane deformation. The C-terminal domain (M1C aa. 165–252) shows no effect on GUV shape and, as previously reported [10], has a low degree of membrane binding (Supplementary Figure S2).

Table 1 (row ‘M1 pH 7.4’) shows a quantitative overview regarding the amounts of deformed GUVs observed for different compositions, in conditions similar to those relative to the samples shown in Figure 1. In order to clearly detect protein binding and, thus, include in the quantification only larger GUVs (> ∼10 μm diameter) that displayed a significant amount of bound M1, we increased the protein concentration to 10 μM (18 μM for GUVs with only 10 mol% DOPS). Visual inspection of several vesicles confirmed that membrane deformation is facilitated by higher DOPS concentrations. A quantitative characterization of M1 binding to deformed membranes is described below (‘M1 exhibits reduced dynamics and high degree of multimerization in deformed vesicles’ section).

**Table 1:** Quantitative characterization of the amounts of non-spherical GUVs for different membrane compositions

	Relative amounts of deformed GUVs				
GUV composition DOPC + DOPS	DOPS 10 mol%	DOPS 20 mol%	DOPS 30 mol%	DOPS 40 mol%	DOPS 50 mol%
M1	0%	24%	49%	83%	89%
pH 7.4	(*n*=29)	(*n*=33)	(*n*=81)	(*n*=84)	(*n*=26)
M1 + PHE			8%	13%	20%
pH 7.4			(*n*=26)	(*n*=23)	(*n*=15)
M1	0%		12%	17%	
pH 5	(*n*=46)		(*n*=25)	(*n*=23)	

Percentages of deformed GUVs are reported for different experimental conditions discussed in the text. The row ‘M1 pH 7.4’ refers to the conditions described for the experiments shown in Figure 1. The row ‘M1 + PHE pH 7.4’ refers to the experiments described in the context of Figure 5A. The observations reported in this table were performed by treating M1-Alexa488 with PHE concentrations between 75 and 150 μM. The row ‘M1 pH 5’ refers to the experiments described in the context of Figure 5B. The total numbers of observed GUVs (*n*) refer in all cases to GUVs that were clearly labeled with fluorescent M1 and had a diameter > ∼10 μm. GUVs that did not display clearly recognizable M1-Alexa488 binding were not considered. The concentration of M1-Alexa488 was 10 μM, with the exceptions of the DOPS 10 mol% (18 μM) and DOPS 50 mol% (6 μM) samples. The percentages summarize the results of at least two independent experiments.

### M1-induced membrane bending in LUVs observed at high resolution

**Figure 2:**
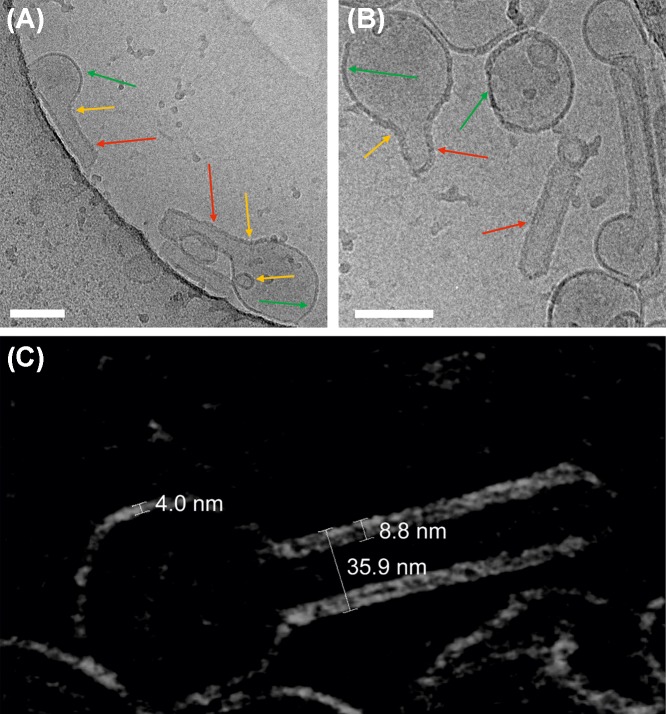
M1 modifies the curvature of LUV membranes (**A,B**) Typical cryo-TEM images of liposomes composed of 40 mol% DOPS in the presence of 10 μM M1 (phosphate buffer pH 7.4, NaCl ∼120 mM, see ‘Materials and methods’ section). The arrows in panels (A,B) indicate protein-free membrane portions of vesicles (green arrows) or M1-bound portions of the bilayer which appear thicker (red arrows: tubules, yellow arrows tubule necks and inward vesiculation), due to the presence of bound M1. Scale bars are 100 nm. (**C**) Cryo-ET of a typical M1-bound liposome (tomography series ±60° at 2° angular increment): the image shows a 15-nm thick section through the three-dimensional volume just in the middle of an LUV incompletely bound to M1 (note the inverted contrast of the so-called voltex representation, i.e. lipid- and protein densities appear light). The numbers indicate the thickness of the bare lipid bilayer (∼4 nm), lipid bilayer with bound M1 (∼8–9 nm) and the diameter of a tubule originating from a vesicle (∼36 nm).

Characterization of bilayer deformation in GUVs via confocal LSM is limited by optical resolution. In order to obtain high-resolution information regarding the interplay between M1 binding and membrane curvature, we used cryo-TEM to observe LUVs in the presence of M1. Lipid vesicles containing DOPC and 40 mol% DOPS were incubated with 10 μM (unlabeled) M1 before freezing. The representative results shown in Figure 2A,B indicate that M1 binds to a large fraction of the lipid bilayers. By observing the apparent bilayer thickness, it is possible to distinguish regions of the bilayer with bound M1 (red and yellow arrows) from those devoid of protein (green arrows). In order to obtain a better insight into the conditions at the membrane, we have employed cryo-ET. Slices of the final 3D reconstruction, calculated from tilting series of images of the LUV embedded in vitreous ice, provide a more accurate representation of membrane spatial features, compared with individual cryo-TEM projection images. Figure 2C shows such a 15-nm thick section through the three-dimensional volume just in the middle of a LUV partially covered by the M1 protein. The protein-free bilayer has a thickness of approximately 4 nm. Regions in which a layer of M1 appears to be bound to the lipid bilayer are between 8 and 9 nm thick. Interestingly, the presence of M1 on vesicles is clearly correlated to changes in vesicle shape. Protein-free LUV surface regions display a positive mean curvature (referred to the membrane monolayer exposed to the protein solution) similar to that observed for control samples (Supplementary Figure S3). Membrane regions to which M1 has bound display various spatial features. Most often, we observed outward tubulation, i.e. membrane surfaces with zero Gaussian curvature, as indicated by the red arrows in Figure 2. In other cases, we observed inward vesiculation or membrane regions with negative Gaussian curvature (yellow arrows in Figure 2).

### Lipid domains modulate M1-induced membrane bending

**Figure 3:**
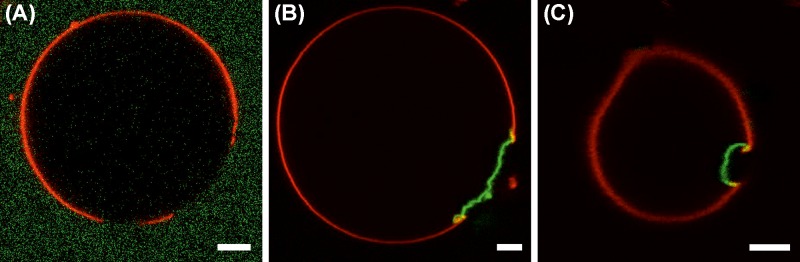
M1 binding to acidic lipid microdomains causes localized membrane deformation (**A**) Typical GUV composed of cholesterol:DPPG:DSPC:DOPC, 10:15:30:45 molar ratios, imaged via confocal LSM. Phase separation was observed by labeling the vesicles with Rhodamine-DOPE 0.01 mol% (red channel, strongly enriched in the disordered phase) and introducing water-soluble Alexa Fluor 488 succinimidyl ester in the outer milieu of the vesicles (green channel). (**B,C**) Typical GUVs with the same compositions as in (A), in the presence of 10 μM M1-Alexa488. The protein is visualized in the green channel and the lateral distribution of Rhodamine-DOPE is represented in the red channel. GUVs similar to that shown in (B) were observed in approximately 75% of the cases. GUVs similar to that shown in (C) (inward budding of the whole ordered domain) were observed in approximately 25% of the cases. All GUVs contained 150 mM sucrose in their lumen and were suspended in a phosphate buffered protein solution (NaCl ∼45 mM, pH 7.4) with similar osmolarity (see ‘Materials and methods’ section). Scale bars are 5 μm. Images were acquired at 23°C.

It was previously reported that IAV assembly and budding occurs in correspondence of confined PM domains [11]. Furthermore, it is known that M1 binds to negatively charged lipids [7,33]. To investigate whether the spatial confinement of acidic lipids within membrane domains can modulate M1-induced membrane deformation, we produced GUVs displaying phase separation in ordered domains (i.e. bilayer regions characterized by highly ordered lipid acyl chains, enriched in saturated phosphatidylcholine and, reasonably, saturated phosphatidylglycerol) and disordered domains (enriched in unsaturated phosphatidylcholine). We have produced similar (supported) lipid bilayers in the past by using a mixture of DOPC, DSPS, DSPC and cholesterol [34]. When producing GUVs, we have noticed that exchanging the saturated phosphatidylserine (i.e. DSPS) with saturated phosphatidylglycerol (i.e., dipalmitoylphosphatidylglycerol, DPPG) improved the yield of phase-separated GUVs. Figure 3A shows an example of such GUVs observed via confocal microscopy (cholesterol:DPPG:DSPC:DOPC, 10:15:30:45 molar ratios). The red channel refers to the lateral distribution of a fluorescent unsaturated lipid analog (i.e. Rhodamine-DOPE), which strongly partitions into the disordered bilayer phase (plausibly, rich in DOPC). The green channel refers to the distribution of a water-soluble fluorescent dye (Alexa Fluor 488 succinimidyl ester). The presence of the dye in the outer milieu allowed the visualization of the whole GUV shape. Ordered lipid domains (plausibly, rich in DPPG and DSPC) can be thus simply identified by the low-partition of Rhodamine-DOPE and appear as dark membrane regions.

After addition of 10 μM M1-Alexa488, approximately half the vesicles showed deviations from spherical shape. More in detail, we observed in certain cases M1-Alexa488 (green channel) bound to irregularly shaped ordered domains (approximately 75% of the cases, typical domain size was approximately 20 ± 10 μm), as shown, e.g. in Figure 3B. In other instances, we observed M1-Alexa488 bound to smaller ordered domains which were budding inward (approximately 25% of the cases, typical domain size approximately 4 ± 1 μm), as shown, for example in Figure 3C. In agreement with previous observations [35,36], we noticed that ordered domains sometime protruded outward (see e.g. Supplementary Figure S4A), independently of the presence of the protein. Finally, in the vast majority of cases, M1-Alexa488 appeared to be excluded from the lumen of the vesicles (see e.g. Supplementary Figure S4B,C for enhanced contrast versions of Figure 3B,C).

### Membrane deformation is accompanied by the formation of a stable M1–M1 network

**Figure 4:**
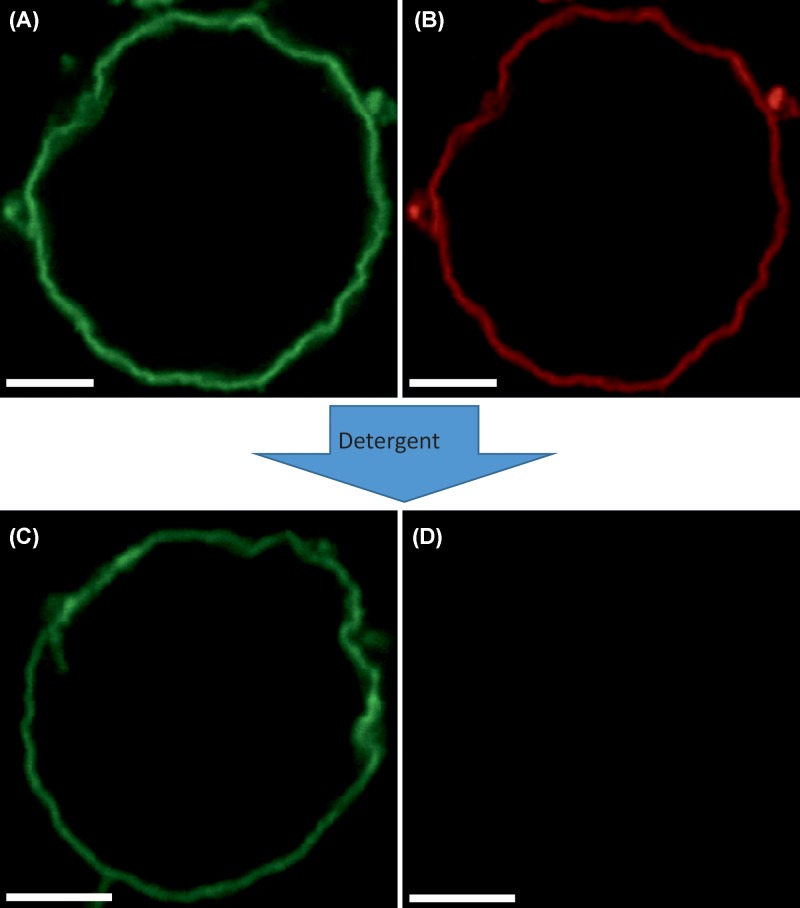
M1 layer interacting with the membrane is stable even after lipid removal (**A,B**) LSM confocal image of a typical GUV composed of DOPC:cholesterol:DOPS 50:20:30, after incubation with 5 μM M1-Alexa488 (green channel, (A)). The lipid bilayer is visualized via the addition of 0.05 mol% Rhodamine-DOPE (red channel, (B)). (**C,D**) LSM confocal image of a (different) typical GUV after treatment for 5 min with detergent (e.g. Triton X-100 1.7 mM). (C) Represents the signal from M1-Alexa488. (D) Represents the signal from Rhodamine-DOPE. The excitation laser power used to acquire the image shown in (D) was approximately seven-times higher than the power used to acquire the image shown in (B). All GUVs contained 150 mM sucrose in their lumen and were suspended in a phosphate buffered protein solution (NaCl ∼25 mM, pH 7.4) with similar osmolarity (see ‘Materials and method’ section). Scale bars are 5 μM. Images were acquired at 23°C.

So far, we have shown that M1–lipid interactions appear to cause alterations in the spatial organization of the bilayer. We next investigated whether M1–M1 interactions are also involved in the membrane deformation process. Figure 4A,B shows a typical GUV containing 30 mol% DOPS in the presence of 5 μM M1-Alexa488. As expected, within 30 min of incubation, deformed vesicles could be observed by monitoring the spatial distribution of a fluorescent lipid analog (Figure 4B) or the labeled protein itself (Figure 4A). It is worth noting that the observed alterations in membrane shape are specific to M1. We have verified that extensive binding of another protein with high affinity to phosphatidylserine (i.e. Annexin V) does not cause significant membrane deformation (see Supplementary Figure S5).

We then proceeded to dissolve the GUVs, by adding 1.7 mM Triton X-100. Most of the lipids were effectively dissolved by the treatment, as demonstrated by the strong decrease in the fluorescence signal of the lipid analog (Figure 4D, for an exemplar GUV). Interestingly, we observed that the protein was not affected by the detergent treatment, as it formed an apparently stable lipid-free three-dimensional structure (Figure 4C). We obtained similar results if lipids were dissolved using a mixture of different detergents (Triton X-100 1.7 mM, n-Dodecylphosphocholine 1 mM, CHAPS 10 mM and n-Dodecyl-β-Maltoside 1 mM).

### M1–M1 interactions are needed for membrane deformation

**Figure 5:**
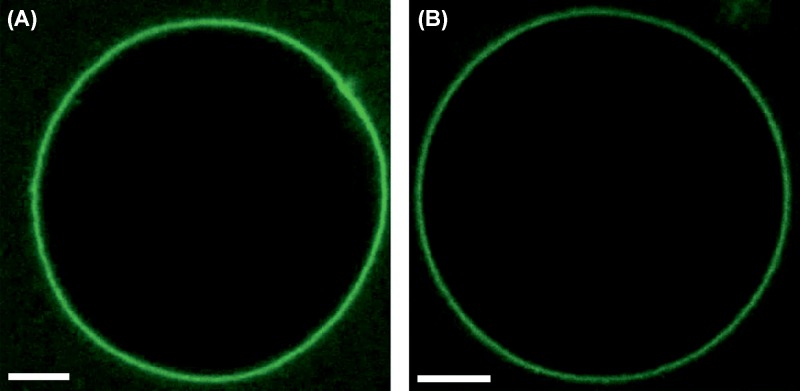
M1-induced membrane deformation requires M1 multimerization (**A**) Confocal LSM image of a typical GUV composed of 40 mol% DOPS and 60 mol% DOPC, in the presence of 10 μM fluorescent M1-Alexa488, which was pre-incubated with 100 μM PHE for 30 min. GUVs contained 150 mM sucrose in their lumen and were suspended in a phosphate buffered protein solution (NaCl ∼45 mM, pH 7.4) with similar osmolarity (see ‘Materials and methods’ section). (**B**) Confocal LSM image of a typical GUV with the same composition as in (A), at pH 5, in the presence of 10 μM M1-Alexa488. These GUVs contained 150 mM sucrose, 10 mM sodium acetate buffer solution at pH 5 in their lumen. The external solution consisted of 10 μM M1-Alexa488 in ∼15 mM sodium acetate buffer (pH 5) with ∼70 mM NaCl and ∼30 mM sucrose (i.e. slightly hyperosmotic conditions, see ‘Materials and methods’ section). Scale bars are 5 μm. Images were acquired at 23°C.

The previous results suggested that M1 forms a protein network around GUVs that remains stable even after lipid removal. This observation hints at the presence of significant inter-protein interactions. To verify whether such M1–M1 interactions have a specific role in altering membrane curvature, we investigated conditions that inhibited protein multimerization, while not completely abolishing membrane binding. First, we incubated GUVs containing 40 mol% DOPS in the presence of 10 μM M1-Alexa488 pre-treated with 100 μM PHE. PHE is a small molecule that disrupts M1–M1 interactions, via direct interaction with the protein [37]. The abovementioned DOPS and M1 concentrations were chosen so that a strong deformation of the GUV bilayer would have been expected (compare with e.g. Figure 1). Strikingly, most of the GUVs appeared spherical in the presence of PHE, as shown in Figure 5A. The same observation was made also under slightly different conditions (i.e. DOPS concentrations between 30 and 50 mol% and M1-Alexa488 incubated with 75–150 μM PHE, see Table 1).

Second, we incubated GUVs containing DOPC and 40 mol% DOPS in the presence of 10 μM M1-Alexa488 at pH 5. Low pH was already shown to interfere with M1 multimerization [9,30,38–42]. Once again, we did not observe significant alterations in the shape of the GUVs (see e.g. Figure 5B). The same effect was observed for GUVs containing 30 mol% DOPS (see Table 1 for a quantitative summary).

### M1 exhibited reduced dynamics and high degree of multimerization in deformed vesicles

**Figure 6:**
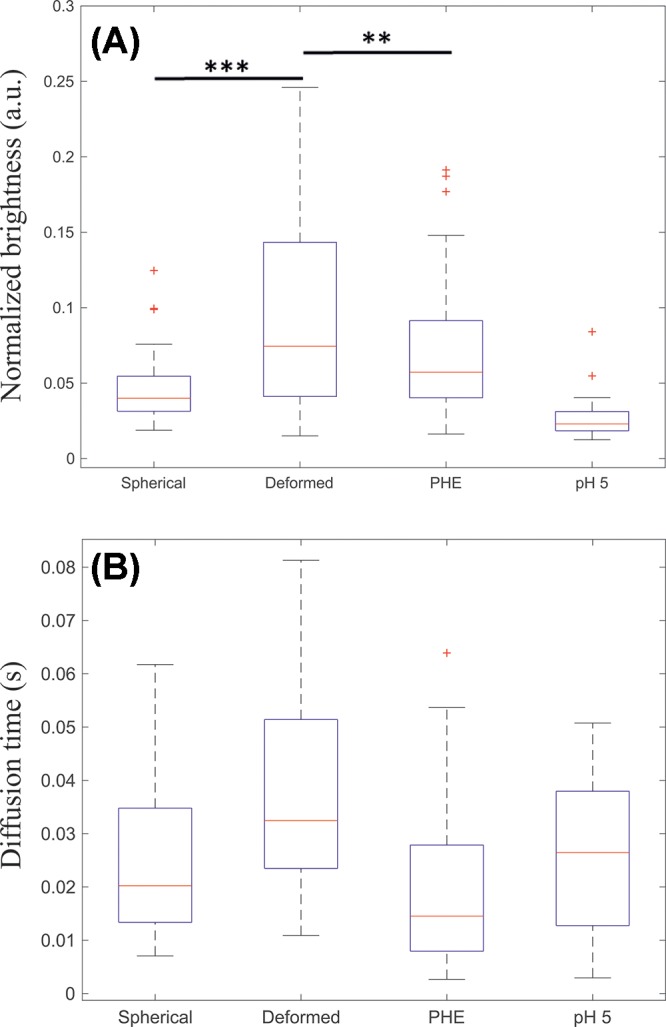
sFCS analysis of M1 brightness and dynamics in spherical and deformed GUVs (**A,B**) GUVs composed of 30 mol% DOPS and 70 mol% DOPC were incubated with 10 μM M1-Alexa488. The categories ‘Spherical’ and ‘Deformed’ refer to measurements in GUVs from samples at pH 7.4 (NaCl ∼45 mM), in the absence of PHE. In these conditions, ∼50% of the GUVs are clearly non-spherical (see Table 1). The category ‘PHE’ refers to spherical GUVs in samples prepared at pH 7.4 (NaCl ∼45 mM), using 100 μM PHE. In these conditions, ∼90% of the GUVs are clearly spherical (see Table 1). The category ‘pH 5’ refers to spherical GUVs in samples prepared at pH 5 (NaCl ∼70 mM), in the absence of PHE. In these conditions, ∼90% of the GUVs are clearly spherical (see Table 1). Details of sample preparation are described in the ‘Materials and methods’ section. sFCS measurements were performed on 16–34 GUVs from two independent sample preparations and the results were pooled together. Each measurement provided M1-Alexa488 normalized brightness values (shown as box plot in (A)), diffusion times (shown as box plot in (B)) and normalized fluorescence intensities (shown in Supplementary Figure S6). Upper outliers from the ‘Deformed’ category are not included in the plot. ‘***’ indicates statistical significant difference between categories, with a *t* test probability outcome *P*<0.01. ‘**’ indicates statistically significant difference between categories, with a *t* test probability outcome *P*<0.05. In the case of diffusion time measurements (B), the category ‘Deformed’ is significantly different from all the other categories, with a *t* test probability outcome *P*<0.01, in all cases.

The results described in the previous paragraphs strongly suggest that M1 multimerization plays a determinant role in causing bilayer deformation. As an alternative approach to characterize protein–protein interactions, we performed sFCS measurements on protein- bound GUVs. We have used in the past similar fluorescence fluctuation analysis methods to characterize M1 multimerization upon interaction with lipid bilayers [9]. In this study, we used sFCS to directly quantitate protein binding, dynamics and multimerization in spherical or deformed GUVs. It is worth noting that the sFCS approach (compared with e.g. point-FCS) is particularly suitable to investigate non-supported membranes, such as GUVs or the cellular PM. We incubated GUVs containing 30 mol% DOPS with 10 μM M1-Alexa488, in different conditions. First, we compared the properties of M1 bound to spherical or deformed GUV within a sample prepared at pH 7.4 (these measurements are referred to as ‘Spherical’ and ‘Deformed’, respectively). As reported in Table 1, such samples contain in average ∼50% deformed GUVs. Furthermore, we have performed sFCS on M1-Alexa488 bound to spherical GUVs in samples that were treated with the M1 multimerization inhibitor PHE [37] or that were prepared at pH 5 (these measurements are referred to as ‘PHE’ and ‘pH 5’, respectively). Both conditions are supposed to be characterized by low M1 multimerization and reduced membrane deformation, as reported in the previous paragraph. Figure 6A shows, for the abovementioned experimental conditions, the measured molecular brightness, i.e. the fluorescence signal detected for each diffusing object per unit of time. Considering that, in a very simple approximation, a fluorescent n-mer contains n-times more fluorescent labels than a monomer, the molecular brightness scales linearly with the size of the fluorescence multimers (e.g. a dimer would have a molecular brightness two times larger than the one of a monomer). In other words, the molecular brightness can be used here to estimate the clustering degree of membrane-bound proteins [9,10]. A normalization procedure was carried out to take into account day-to-day variations in protein labeling efficiency and in the experimental setup (see ‘sFCS’ section). Nevertheless, a conversion from molecular brightness into a precise multimeric state is, in this case, not straightforward. First, M1 is probably present as an undefined mixture of different multimeric species (and sFCS will detect a weighted average of the different multimeric species). Second, the low degree of protein labeling (∼0.1 label/protein ratio) implies that the effective number of fluorescent molecules in multimers of different sizes will be very similar. For example, fluorescent monomers and the vast majority of fluorescent dimers will both contain exactly one fluorescent label. This effect can be accounted for, as described by Dunsing et al. [31]. Third, the brightness value of a monomeric reference would be required (i.e. a monomeric molecule labeled with the same fluorescent probe and diffusing in a system with the same geometrical properties as M1-Alexa488) [26]. In this context, using the same fluorescent label diffusing in solution or bound to a different (monomeric) protein on the membrane would not be a precise reference in general, due to the different geometry [43] and possible changes in quantum yield of the probe, respectively. Nevertheless, some simplifications can be made in order to estimate the multimerization variations among the different samples. In the simple approximation that M1 is present (i) as a single multimeric species and (ii) in dimeric state at pH 5 [30], we could estimate that M1 forms approximatively decamers in spherical vesicles, 25-mers (and up to 100-mers) in deformed vesicles, and 15-mers in samples treated with PHE. These estimations take into account the degree of protein labeling, as described in ‘sFCS’ section. In summary, our brightness measurements indicate that M1 bound to deformed vesicles (at pH 7.4, in the absence of PHE) is characterized by a significantly higher degree of multimerization, compared with the protein bound to spherical GUVs within the same samples. Also, both lower pH and the presence of the multimerization inhibitor PHE resulted in significantly decreased M1 multimerization, as expected.

Additionally, sFCS provided quantitative information about M1-Alexa488 average diffusion dynamics. More precisely, we report the typical time needed by the protein to diffuse through a membrane area intersected by the detection volume. Such diffusion time is inversely proportional to the Brownian diffusion coefficient. The results shown in Figure 6B indicate, as expected, that M1 dynamics in deformed vesicles is slower than that observed in all other cases. It is worth noting that protein diffusion is, in general, dependent on both the size of protein assemblies and protein–membrane interaction.

In conclusion, these data indicate that M1-induced membrane deformation is clearly accompanied by a significant increase in protein multimerization and reduced protein dynamics. On the other hand, we did not observe a simple correlation between total amount of bound protein and the induction of membrane deformation, as the total fluorescence signal measured in deformed or spherical GUVs was similar (see Supplementary Figure S6).

## Discussion

The protein M1 is believed to play a fundamental role in the assembly of IAV [6]. In order to release a new virion from the PM of an infected cell, the lipid bilayer has to undergo a shape change. In particular, some viral or cellular component(s) must induce a negative curvature on the inner leaflet of the PM, so that a virion can bud out from the cell. Previous studies have suggested that different viral proteins might be responsible for the reshaping of the PM [11–17]. M1 has also been suggested to be capable of inducing curvature in the bilayer, but no unequivocal evidence has been presented yet. While investigations *in cellulo* provide information in a biologically relevant context (e.g. [12,13]), the concurrent presence of several cellular and viral proteins does not allow the isolation and characterization of specific protein-protein or protein–lipid interactions. For this reason, we have applied here a *bottom-up* approach and modeled the interaction between M1 and the PM in a controlled environment. In particular, we have characterized the interaction between M1 and physical models of the PM (i.e. GUVs or LUVs) using several microscopy methods. A similar approach has been recently used to investigate the interaction between the matrix protein from the Influenza C virus and lipid membranes [22]. It was shown that this specific matrix protein forms elongated structures on GUVs and can also cause the formation of lipid tubules (by inducing negative membrane curvature). In spite of significant similarities in tertiary structure, the low sequence similarity between the two proteins and the morphologically different membrane formations observed in cells infected by the two viruses [44,45] do not allow extending the conclusions regarding the matrix protein of Influenza C directly to Influenza A M1.

In the current work we have investigated for the first time the interaction between IAV M1 and GUVs containing different amounts of DOPS. The protein concentration (much larger than the reported Kd values [10,39]) was chosen so to take into account that, *in vivo*, viral proteins might be recruited to small PM domains [46] and, therefore, reach a high local concentration [47]. Previous investigations on solid-supported bilayers have demonstrated that M1 binds to lipid membranes containing negatively charged lipids and that M1–lipid binding is accompanied by extensive protein multimerization [9]. In agreement with these observations, we have observed that M1 binds effectively to GUVs containing DOPS. Surprisingly, M1 binding induced a significant alteration in the shape of the vesicles, especially at higher phosphatidylserine concentrations (e.g. >30 mol%, see Figure 1). Specifically, the N-terminal domain of M1 is sufficient to induce membrane shape changes, in line with previous findings suggesting that this domain of the protein is responsible for M1–lipid and M1–M1 interactions [7,10]. In contrast, the N-terminal domain of Influenza C M1 is not sufficient to cause membrane curvature [22]. Observation via optical microscopy indicates that M1 binds homogeneously while altering the spherical shape of vesicles. In general, both regions with positive and negative curvature can be observed. It is reasonable to assume that GUVs might become (temporarily) unstable, due to the significant and extensive deformation and volume decrease, thus allowing the internal solution to leak out. Accordingly, we have observed that M1 might indeed penetrate into the lumen of several vesicles and thus bind to both membrane leaflets. Nevertheless, reproducible membrane deformation patterns are observed also in the cases in which the protein seems to bind only to the outer leaflet (Supplementary Figure S1 or, in the case of spatially limited deformations, Figure 3 and Supplementary Figure S4). Alterations in bilayer curvature were also observed if M1 was incubated with LUVs with diameter ∼100 nm. Thanks to the high spatial resolution of cryo-TEM and cryo-ET, M1-lipid interaction could be observed with remarkable detail. On a nanometer scale, binding of the protein to the vesicles appears not homogeneous. M1 seems capable to induce tubulation and, in fewer cases, vesiculation. The protein is found also concentrated in membrane regions with negative Gaussian curvature (i.e. at the neck of tubes) (Figure 2). While comparing these results with the possible membrane-bending properties of M1 in a physiological context, it must be kept in mind that LUVs are more curved compared with GUVs or the PM. The specific protein–lipid arrangements which are observed for LUVs (e.g. approximately 20 nm radius observed for tubular structures) might therefore also be influenced by the initial membrane curvature. Furthermore, the protein–lipid ratio used in the present study for GUV samples was at least ten-times lower than the one used for LUV samples, and this difference might play a role in the formation of protein–lipid structures. Finally, buffer concentrations in GUV and LUV samples were slightly different. Nevertheless, taken together, the experiments performed in GUVs and LUVs suggest that M1 is sufficient to induce membrane deformation. While no other membrane component appears necessary for M1-induced membrane bending, the possibility that e.g. other viral proteins might modulate this effect (by, e.g. making negative curvature of the leaflet interacting with M1 energetically favorable over positive curvature) is currently under investigation. Furthermore, it is worth mentioning that M1 was reported to form various multimeric arrangements (e.g. helical [41]) that, *in vivo*, might result in a different remodeling of lipid membranes, compared with the one presented in the current work. Lipid ordering in the bilayer does not appear to play a dominant role in M1-induced membrane bending, since we observed no effect induced by altering membrane fluidity (by increasing cholesterol content [48] or in the presence of saturated lipids). We further noticed that M1-induced deformation does not require low membrane lateral tension, as it occurs also in hypoosmotic conditions (Supplementary Figure S5C,D). Additionally, our observation that significant shape alteration is observed only in bilayers containing higher amounts of DOPS is likely not connected to an effect of this lipid on the physical properties of the membrane. It was suggested in fact that phosphatidylserine does not decrease the bending stiffness of a lipid bilayer [49,50]. In conclusion, the observed alterations in bilayer shape are brought about specifically by the binding of M1 to the membrane. Similar results are observed if M1 binds to GUVs via interactions with other lipids (i.e. in the absence of phosphatidylserine), such as PG, PIP2 or metal-ion-chelating lipids binding the His-tag of M1 (data not shown).

A further aspect that was examined in these experiments regarded the lateral organization of the bilayer, prior to protein binding. Previous investigations have shown that M1 multimerization and binding to lipid membranes are modulated by the presence of lipid domains containing negatively-charged lipids [34]. In infected cells, M1 clusters are observed in correspondence of phosphatidylserine-rich membrane regions [34]. In order to clarify whether M1-induced membrane restructuring is also affected by the lateral organization of the lipid bilayer, we investigated lipid mixtures displaying phase separation. In particular, GUVs contained ordered domains (enriched in cholesterol, saturated lipids including, reasonably, negatively charged saturated lipids) in a disordered bilayer (enriched in unsaturated lipids). In the presence of M1, the ordered domains showed variations from the original spherical shape and, especially in the case of smaller domains, inward budding (see e.g. Figure 3B,C). This observation demonstrates, on one hand, that these ordered domains are indeed enriched in acidic lipids to which M1 can effectively bind. Second, it indicates that the membrane restructuring effect of M1 appears significant even at relatively high values of membrane bending rigidity (cfr., for example, approximately 65 kT for a DPPC:cholesterol 80:20 bilayer [51]). Third, it suggests that the local membrane restructuring induced by M1 might be modulated by the lateral lipid organization in the bilayer region in which M1 is concentrated. This phenomenon might be relevant *in vivo*, since M1 is supposed to be confined in budozones, i.e. small domains of the PM of infected cells from which IAV budding takes place [52].

Finally, we aimed to clarify the molecular mechanism driving the deformation of the membrane induced by M1. Several mechanisms for the induction of membrane curvature driven by (non transmembrane) proteins were described, including: membrane insertion, protein crowding and scaffolding [53–56]. In order to distinguish among these possibilities, we observed M1–GUV interaction in conditions in which M1 could bind to the membrane but protein multimerization was hindered (i.e. low pH or in the presence of a multimerization inhibitor, see Figure 5). These experiments revealed that protein binding to the bilayer *per se* did not induce significant alterations in membrane shape. The reduced ability of M1 to form large multimers in these conditions was confirmed via sFCS measurements (Figure 6). Accordingly, the amount of M1 bound to deformed vesicles was not significantly higher than that of M1 bound to spherical vesicles (Supplementary Figure S6). Finally, we verified that binding of another protein to DOPS in GUVs (in comparable amounts with M1) did not affect membrane shape (Supplementary Figure S5). Taken together, these observations suggest that membrane insertion (assuming that protein insertion is indeed not altered by low pH or PHE treatment) or protein crowding might not be the main factors driving M1-induced curvature. On the other hand, we observed that M1 forms a protein network which is characterized by slow dynamics (Supplementary Figure S5 and Figure 6B) and seems to impose its irregular (corrugated) shape on the underlying bilayer. The strength of M1–M1 interactions allows the protein shell to remain stable even after removal of the lipid membrane using detergents (Figure 4). Additionally, we were able to quantitatively verify that M1 bound to deformed vesicles forms in general larger multimers (up to approximately ten-fold), compared with the case of spherical vesicles (Figure 6). In this regard, the limitations of the sFCS approach in this context should be mentioned: First, the reported brightness/multimerization and diffusion values refer to an average of different multimeric species that might be present in the sample. Second, the presence of an immobile protein fraction would not be detected by fluorescence fluctuations techniques which, in general, report only of the properties of diffusing molecules. Third, membrane geometry and the detection area are not well-defined in the case of deformed membranes with large curvature (compared with the typical size of the detection volume of ∼300 nm). In other words, a larger than expected bilayer surface (due to ruffling within the detection volume) might be observed during our experiments. As a consequence, protein diffusion times and total fluorescence intensity might be overestimated in deformed vesicles. On the other hand, protein brightness and multimerization would be underestimated. Of interest, these limitations do not affect our main findings that: (i) the amount of M1 bound to deformed vesicles is not significantly higher than that of M1 bound to spherical vesicles and (ii) M1 bound to deformed vesicles is characterized by a higher degree of multimerization.

In conclusion, our data show that IAV M1 binds to vesicles containing negatively-charged lipids, forming a stable protein layer. This M1–M1 network seems to be characterized by both regions of negative and positive curvature, although it appears that its precise spatial structure might be modulated by the initial curvature of the bilayer and the presence of lipid-protein lateral confinement. Our results support the model according to which the M1 shell, *in vivo*, might drive the IAV budding (likely in concert with other membrane and/or viral components that help determining a specific curvature) and provides mechanical stability to the newly formed virion (see e.g. [57]).

## Supporting information

**Supplementary Figure S1:** Control experiments regarding the effect of labeling and buffer conditions.

**Supplementary Figure S2:** The N-terminal domain of M1 is sufficient to induce membrane deformation.

**Supplementary Figure S3:** Protein-free controls for membrane deformation.

**Supplementary Figure S4:** Phase separated GUVs interacting with M1.

**Supplementary Figure S5:** Comparison of M1 and Annexin V binding. Annexin V is characterized by faster dynamics and does not induce significant membrane deformation

**Supplementary Figure S6:** sFCS analysis of M1 binding to spherical and deformed GUVs.

## Abbreviations

aa: amino acid
CM1: M1 C-terminal domain, 165–252 aa
cryo-ET: cryo-electron tomography
cryo-TEM: cryogenic transmission electron microscopy
DOPC: dioleoyl-sn-glycero-3-phosphocholine
DOPS: 1,2-dioleoyl-sn-glycero-3-phospho-l-serine
DPPG: 1,2-dipalmitoyl-sn-glycero-3-phospho-(1′-rac- glycerol)
DSPC: 2-distearoyl-sn-glycero-3- phosphocholine
GaAsP: Gallium arsenide phosphide
GUV: giant unilamellar vesicle
IAV: influenza A virus
IPTG: Isopropyl beta-D-1-thyogalactopyranoside
ITO: indium tin oxide
LSM: laser scanning microscopy
LUV: large unilamellar vesicle
NM1: M1 N-terminal domain, 1–164 aa
PHE: 2-(4-(3-(4-acetyl-3-hydroxy-2-propylphenoxy) propoxy) phenoxy acetic acid
PIP2: phosphatidylinositol 4,5-bisphosphate
PM: plasma membrane
Rhodamine-DOPE: 1,2-dioleoyl-*sn*-glycero-3-phosphoethanolamine-*N*- Lissamine rhodamine B sulfonyl
sFCS: scanning fluorescence correlation spectroscopy
